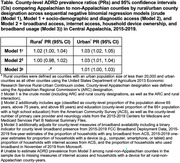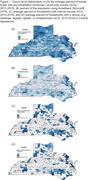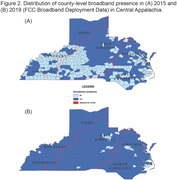# Broadband access may influence variation in Alzheimer’s disease and related dementias prevalence in Central Appalachia

**DOI:** 10.1002/alz.092545

**Published:** 2025-01-09

**Authors:** Jenna Rajczyk, Jeffrey Wing, James Burke, Wendy Yi Xu

**Affiliations:** ^1^ Ohio State University, Columbus, OH USA

## Abstract

**Background:**

Geographical differences in the burden of Alzheimer’s disease and related dementias (ADRD) exist in the US. Rural and Appalachian areas are characterized by limited access to primary and specialty health care in comparison to their urban and non‐Appalachian counterparts. Better access to telehealth can improve detection of ADRD in remote regions but it highly relies on availability of broadband services. Our objective is to evaluate the influence of county‐level broadband availability on Appalachian and rural variation in ADRD prevalence in Central Appalachia.

**Method:**

County‐level ADRD prevalence among fee‐for‐service Medicare beneficiaries in Central Appalachia (Kentucky, North Carolina, Ohio, Tennessee, Virginia, and West Virginia) was estimated using 2015‐2018 Centers for Medicare and Medicaid Services Public Use Files. Broadband availability was defined as county‐level measures of broadband presence (FCC Broadband Deployment Data 2015‐2019), proportion of households with broadband, household device ownership, household internet access (American Community Survey 2015‐2019), and broadband usage (Microsoft 2019). ADRD prevalence by Appalachian Regional Commission’s Appalachian/non‐Appalachian designation, and by rural/urban classification (Rural‐Urban Continuum Codes) was estimated using sequential negative binomial regression models: (1) crude; (2) adjusted for age, education, and care access (PCP and neurology visits); (3) additionally adjusted for all broadband measures.

**Result:**

Among all Central Appalachian counties, 52%‐97% had broadband access throughout the study period (Figures 1‐2). The proportions of urban (61%) and non‐Appalachian counties (60%) with broadband access were higher relative to rural (39%) and Appalachian (40%) counties. Appalachian counties had higher prevalence in both rural (Prevalence Ratio (PR): 1.02; 95% CI: 1.00, 1.04) and urban (PR: 1.03; 95% CI: 1.02, 1. 05) areas, but this was mostly explained by socio‐demographics in rural areas (PR: 1.00, 95% CI: 0.98, 1.02; Table). Variation in urban counties persisted after adjustments for demographics, access, and broadband (PR: 1.01; 95% CI: 1.00, 1.03).

**Conclusion:**

Variation in ADRD prevalence was largely explained by socio‐demographics and care access but not broadband availability. However, the role of broadband access on ADRD prevalence needs to be clarified. Greater broadband access may reflect improved telemedicine access and higher diagnoses, yet the inconsistency in rural counties challenges this hypothesis and other explanations should be considered.